# Regulation of dermal circadian pathways by a novel topical formulation

**DOI:** 10.1111/ics.13017

**Published:** 2024-09-01

**Authors:** Charlene DeHaven, Stephanie Wheeler, Anna Langerveld, C. Bryan Johns

**Affiliations:** ^1^ Innovative Skincare Burbank California USA; ^2^ Genemarkers LLC Kalamazoo Michigan USA

## Abstract

Skin health is impacted by a wide range of intrinsic and extrinsic factors (*J Dermatol Sci*, 2017, 85, 152), including those that impact circadian rhythm, such as sleep disruption (*Textbook of Aging Skin*, 2016), UV (*Biomed Aging Pathol*, 2013, 3, 161) and blue light (*Int J Cosmet Sci*, 2019, 41, 558). Disruption of the skin's endogenous circadian balance, even by a consistently late bedtime, has deleterious effects on multiple measurements of skin health, including hydration, skin barrier protection, microbiome counts and skin regeneration, among others (*Clin Cosmet Investig Dermatol*, 2022, 15, 1051). Skin repair processes occur at night and help to maintain important aspects of skin health (*FEBS Lett*, 2021, 595, 2413). Interest is increasing in the development of topical products that help restore proper circadian function. This study demonstrates that a proprietary topical formulation regulates new and established gene and protein biomarkers of circadian entrainment and circadian rhythm, demonstrating the product's potential to maintain appropriate dermal diurnal balance.

## Graphical abstract

There is increasing interest in the development of topical products that help to restore proper circadian function in the skin, since maintenance of circadian rhythms and entrainment impact skin health. Genetic and immunofluorescence data are used to describe such a topical.

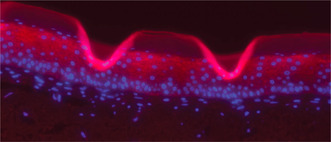

## Résumé

La santé de la peau est affectée par un large éventail de facteurs intrinsèques et extrinsèques (*J Dermatol Sci*, 2017, 85, 152), y compris ceux qui ont un impact sur le rythme circadien, tels que les troubles du sommeil (*Textbook of Aging Skin*, 2016), les UV (*Biomed Aging Pathol*, 2013, 3, 161) et la lumière bleue (*Int J Cosmet Sci,* 2019, 41, 558). La perturbation du rythme circadien endogène de la peau, même par un coucher systématiquement tardif, a des effets délétères sur de multiples mesures de la santé de la peau, notamment l’hydratation, la protection de la barrière cutanée, le nombre de microbiomes et la régénération de la peau, entre autres (*Clin Cosmet Investig Dermatol*, 2022, 15, 1051). Les processus de réparation de la peau se produisent la nuit et aident à maintenir les aspects importants de la santé de la peau (*FEBS Lett*, 2021, 595, 2413). L’intérêt est accru dans le développement de produits topiques qui aident à restaurer une fonction circadienne correcte. Cette étude démontre qu’une formulation topique exclusive régule les biomarqueurs génétiques et protéiques nouveaux et établis de l’entraînement circadien et du rythme circadien, ce qui montre le potentiel du produit à maintenir un équilibre diurne cutané approprié.

## OBJECTIVES

The sleep–wake cycle, also known as the circadian rhythm, is maintained by a homeostatic feedback loop of fluctuating levels of brain activity regulated by the suprachiasmatic nucleus pacemaker (SCN) [1, 2, 3, 4, 5, 6, 7] and neurohormonal mediators, including melatonin [8, 9] and cortisol [10]; in simple terms, melatonin induces sleep and exhibits other properties, while cortisol's multiple skin effects are both related to wakefulness and simultaneously affected by the sleep–wake cycle [11], as well as other factors. The circadian rhythm runs on a roughly 24‐h period synced with the light–dark cycle. In mammals, the circadian clock is expressed in almost all cells, including peripheral tissues such as the skin [12]. While exposure to the light–dark cycle is the primary inducer of entrainment for the skin's peripheral clock, other external stimuli, such as food intake and temperature, can influence circadian processes in skin tissues [2]. The peripheral clock within the skin is also able to adapt to environmental cues. UV and blue light can directly influence the circadian rhythm in the skin [2, 13] via DNA damage and oxidative stress or by inducing melatonin signalling [14].

Melatonin is a hormone that plays a critical role in regulating circadian rhythms and is found in almost all vertebral tissues, including the skin [15], one of the organs in which it is synthesized. In the skin, melatonin has antioxidant effects by scavenging radicals and inhibiting their production, maintaining mitochondrial function, affecting thermoregulation, enhancing stem cell motility in wound healing and producing anti‐inflammatory and anti‐apoptotic effects [16, 17]. Topical melatonin has been shown to be effective in both photoprotection and anti‐ageing formulations [18].

Different types of UV exposure (i.e. UVA, UVB or UVC) have been shown to increase or decrease the expression of different core clock genes, such as CRY2, BMAL1, CLOCK and PER1 [19]. Circadian entrainment has been associated with skin biological processes such as hydration [20], aging and rejuvenation [21], wound healing [22], oxidative stress response [21, 22] and ultraviolet radiation protection [21, 23].

Dysregulation of circadian rhythm in the skin has been linked to multiple skin conditions such as atopic dermatitis and psoriasis [6]. The primary signalling feedback loop of the circadian clock involves the regulation of a cascade of well‐established molecules: BMAL1 dimerizes with CLOCK or NPAS2, and this dimerization triggers the expression of PER, CRY, ROR, NR1D1, DBP and other clock‐controlled genes. After reaching a concentration threshold, PER and CRY dimerize to inhibit BMAL1: CLOCK from binding to DNA [13, 24]. CLOCK and PER1 are expressed in keratinocytes, melanocytes and dermal fibroblasts [20, 25].

There has been interest in the development of topical products that target circadian rhythms, partly due to our greater understanding of the specific molecular mechanisms that regulate circadian rhythm in the skin. In addition to topical product formulation, the timing of the product's application would be an important consideration that can influence the skin's response. For example, the stratum corneum rhythmically displays higher permeability in the evening compared to the morning, meaning topical products may penetrate the skin better when applied at night and in addition, blood flow in the skin increases at night, which may influence absorption [18]. This study aimed to demonstrate that a novel topical formulation containing a proprietary blend of retinol and additional additives can regulate circadian processes in the skin and help restore balance.

## METHODS

### In vitro skin tissue culture

Full‐thickness EFT‐400™ (MatTek®) in vitro skin tissues comprised of normal human keratinocytes in a stratified corneal layer, a dermal component containing an intact dermal–epidermal junction (DEJ), and viable fibroblasts were used for this study. This human skin equivalent model has undergone validation as a reliable research tool [26, 27, 28, 29, 30, 31, 32, 33]. Four cultures were included in each treatment group. A 15 μL volume of test material (TM) was applied to the centre of each culture using a calibrated positive displacement pipette. Following topical applications, cultures were returned to the incubator at 37°C with 5% CO_2_ and ~95% relative humidity for an initial 10‐h exposure. After 10 h, the products were rinsed from the surface of the tissues, and the tissues were left dry until a second application of the material (24 h following the initial treatment). This regimen was repeated one additional time for final tissue collection 48 h after the initial treatment. At the time of collection, the TMs were rinsed off using Dulbecco's phosphate buffered saline (DPBS); the excess liquid was removed with a sterile cotton swab, and tissues were split into two halves; one half was transferred into RNAlater solution, and the other half was transferred into 10% neutral buffered formalin.

An additional set of tissues was maintained and treated as described above but collected at 32, 40 and 48 h post‐initial treatment to confirm RNA‐Seq [34, 35, 36, 37, 38] results and to evaluate gene expression at multiple time points due to the cyclical nature of circadian processes.

The two test materials (TMs), ‘TMX’ and ‘TMY’, consisted of proprietary blends of ingredients with retinol and retinol‐like compounds as key actives. ‘TMX’ and ‘TMY’ were identical formulations with the exception of the retinol concentration (0.3% ‘TMX’, 1.0% ‘TMY’). Both ‘TMX’ and ‘TMY’ were composed of the active ingredients mixed in normal saline. Tissues treated with saline served as the vehicle controls. Saline was considered the most suitable control due to the fact that if all actives were removed from each of the materials in the blended formulations, saline would be the only remaining substance.

### RNA extraction, quantification and qualification

Total RNA extractions were performed using a Maxwell RSC SimplyRNA kit (Promega, WI) with the addition of a Proteinase K incubation. For sequencing, the RNA samples were quantified on a Qubit flex fluorometer (Thermo Fisher Scientific, MA), and 1000 ng of input RNA was used for each library preparation. All RNA samples showed an RNA Integrity Number (RIN) ≥ 8.0. RNA concentration and purity were determined using a Nanodrop 2000 spectrophotometer (Thermo Fisher Scientific, MA) for additional qPCR confirmation of select targets.

### Library preparation and sequencing

Poly(A) RNA sequencing libraries were prepared following Illumina's Stranded‐mRNA library preparation (Illumina, CA) protocol, with the exception of additional drying times (5 min) for ethanol in the clean‐up steps. The sequencing library's quality control (QC) analysis was performed using DNA 1000 and High Sensitivity DNA Chips on an Agilent Bioanalyzer instrument. Single‐ended sequencing was performed on Illumina's Next‐seq 550 Dx sequencing system to achieve a minimum read depth of 10 million reads per sample.

### Differential gene expression analysis

Differential gene expression analysis was conducted using several apps in Illumina's BaseSpace sequence hub. The BCL convert app converted the BCL files from the sequencer into FASTQ format. The DRAGEN Differential Expression application v 4.0.3 was used to run the DESeq2 algorithm on RNA quantification files produced by the DRAGEN RNA app. This identifies genes and transcripts that are differentially expressed between the treatment group and control group. The differentially expressed genes (DEGs) were identified using an adjusted *p*‐value cut off of <0.05 and linear fold change cut‐off of >1.5 or <−1.5 (Log_2_FC > 0.6 or <−0.6).

### Functional enrichment and pathway analysis

Tertiary RNA‐Seq data analysis was performed using the Database for Annotation, Visualization, and Integrated Discovery (DAVID) (https://david.ncifcrf.gov). The DEGs identified between the treatment and control groups were selected for gene ontology (GO) enrichment and Kyoto Encyclopedia of Genes and Genomes (KEGG) pathway analyses. Following a separate enrichment analysis strategy, up and downregulated genes were uploaded separately to DAVID for pathway enrichment.

Additional analysis was performed for two pathways of interest related to circadian genes: Circadian Entrainment and Circadian Rhythm (KEGG pathway IDs hsa04713 and hsa04710, respectively). The analysis of these two pathways was utilized to discern similarities and differences between the two test materials (‘TMX’ and ‘TMY’) and their molecular mechanisms of action. Briefly, KEGG diagrams for the two pathways were reviewed for the relationships between each of the genes regulated by either or both TMX and TMY (i.e. which genes were upstream or downstream of each other, which genes were up or downregulated by each material and which genes were shared/unique to each material).

### Immunofluorescence

The tissues preserved in 10% formalin were sent to the histology core facility at Western Michigan University for immunofluorescence (IF) staining of the target proteins. Three 5‐μ sections from each tissue were stained for each protein target. Positive and negative tissue control sections were included for each antibody marker and reagent negative control sections (excluding the primary antibody step). Antigen retrieval was performed using the Epredia PT module standard protocol 98°C for 20 min with retrieval solution Dewax and HIER L Buffer (Epredia, TA‐999‐DHBL). Specimens were incubated with the primary antibody for 1 h, followed by a linker and then secondary antibody reagent for 10 min each, all at room temperature, then coverslipped. Digital images of the stained slides were captured between 10× and 40× magnification using a Nikon Eclipse Ti2‐E Inverted Microscope with NIS Elements AR Software.

Images were quantified using ImageScope software. Briefly, NIS Elements was used to convert the raw images into TIFF files. TIFF files were analysed in ImageScope. ImageScope was used to calculate area, integrated intensity and mean grey value for each image's region of interest (ROI) and background areas. These measurements were utilized to calculate the corrected total cell fluorescence (CTCF).

$$\text{CTCF}=\text{Integrated Density}-\left(\text{Area of selected}\;\mathrm{ROI}\times \text{Mean Fluorescence of background readings}\right)$$

Three sections of each tissue were analysed, and the mean CTCF per sample was calculated. The mean CTCF was also calculated per biological treatment group (*n* = 4).

### cDNA synthesis and qPCR processing

cDNA was generated using a High‐Capacity cDNA Synthesis kit according to the manufacturer's instructions (Applied Biosystems). cDNA was generated from 1000 ng RNA per sample. qPCR reactions were run using validated TaqMan® gene expression assays in a 384‐well format. Assays were run in a Life Technologies QuantStudio 12k Flex instrument. Each gene was assayed in duplicate.

### qPCR data analysis

qPCR data quality and statistical analysis were assessed and performed on the raw data files using ThermoFisher Connect Software (Life Technologies). Statistical analysis was performed using the relative quantitation (RQ) method and unpaired *t*‐tests. In the first step of an RQ analysis, the Cq value of the target gene is normalized to the Cq value of an endogenous control gene to generate the delta Cq (dCq). dCq values are calculated in order to normalize variability between the samples that may occur during the experimental procedures. The statistical software converts the dd Cq values into log and linear RQ values for export [RQ = 2‐ddCq]. The linear RQ values were converted to linear fold‐change values to simplify data interpretation; linear fold‐change data were calculated from exported linear RQ values using Microsoft Excel.

## RESULTS

### Differential gene expression via RNA‐seq

**FIGURE 1:**
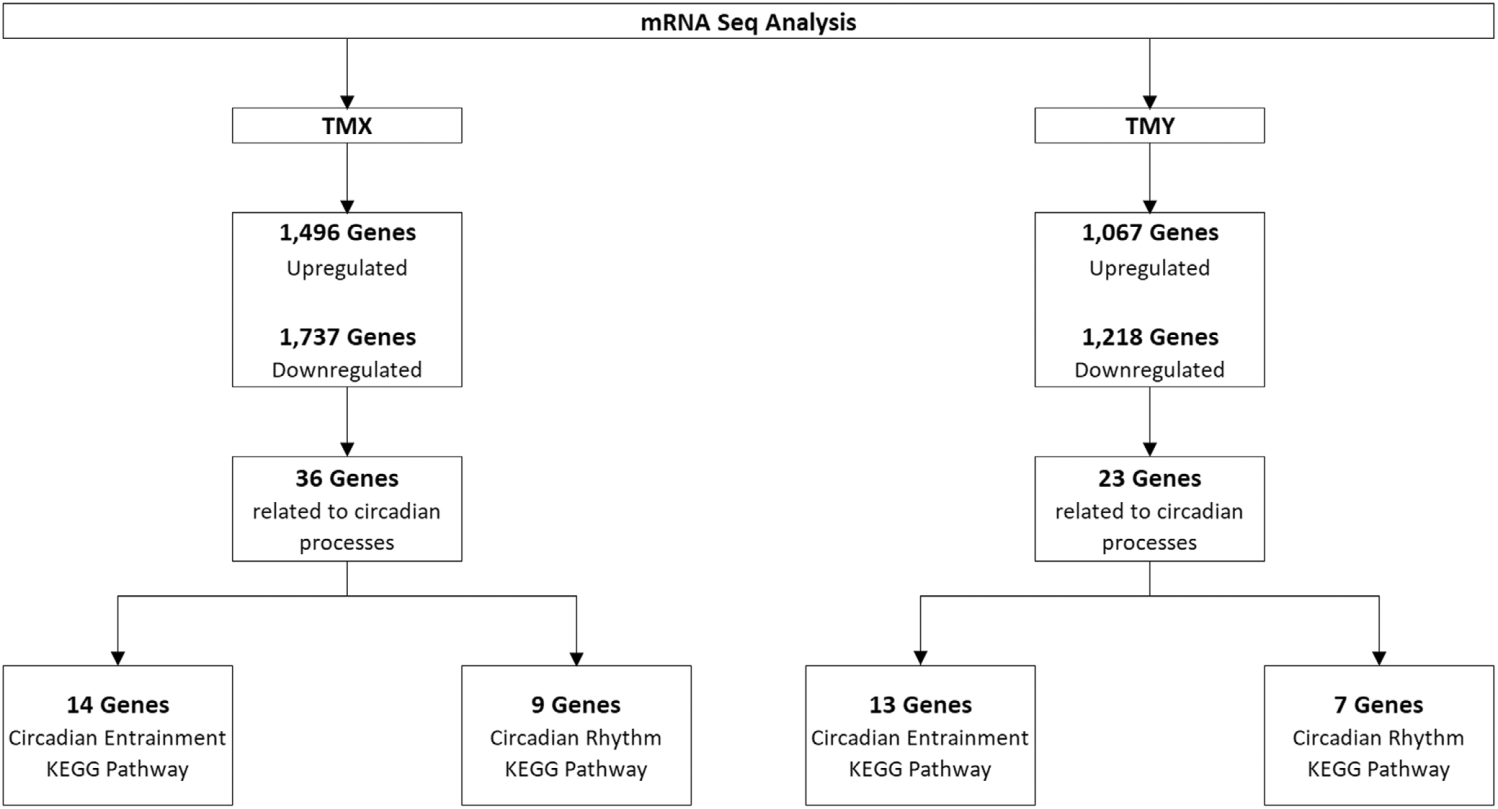
Summary of differentially expressed genes.

‘TMX’ significantly (adjusted *p* < 0.05) regulated (linear fold‐change ≥1.5) 3233 differentially expressed genes (DEGs), with 1496 upregulated and 1737 downregulated. ‘TMY’ significantly regulated 2285 DEGs, with 1067 upregulated and 1218 downregulated. Of the regulated genes, ‘TMX’ regulated 36 genes related to circadian processes, while ‘TMY’ regulated 23 genes. Of the circadian‐related genes, 23 genes for ‘TMX’ and 20 genes for ‘TMY’ were directly involved in one of two KEGG pathways (Figure 1).

**FIGURE 2:**
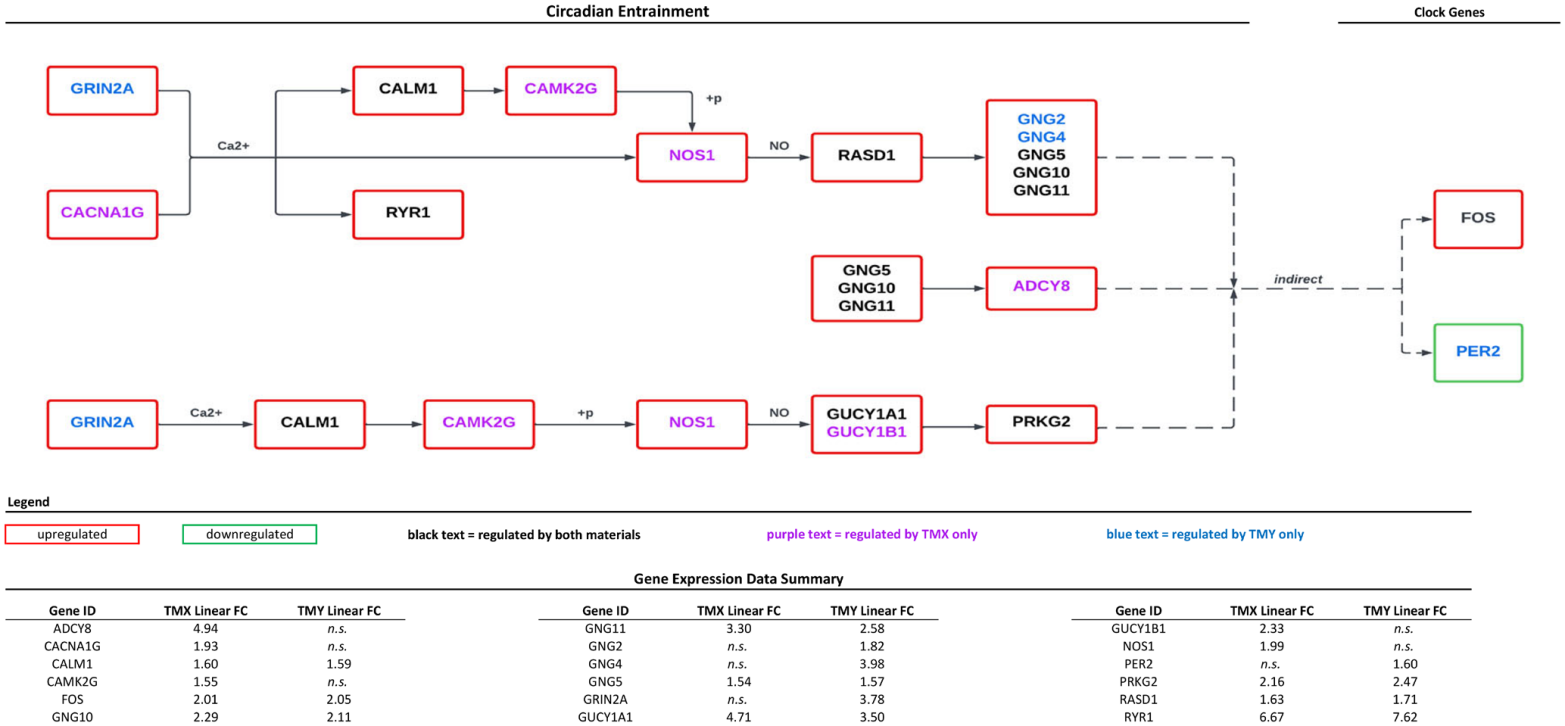
Circadian entrainment. Image adapted from KEGG submission for #04713, Kanehisa Laboratories, 28 January 2019. Only genes regulated in this study are shown; n.s., not significant. [Colour figure can be viewed at wileyonlinelibrary.com]

Additional analysis was performed for the KEGG pathways Circadian Entrainment (#04713) and Circadian Rhythm (#04710). For the Entrainment pathway, both materials significantly regulated nine genes, with ‘TMX’ regulating five unique genes and ‘TMY’ regulating four genes. All genes in this pathway were upregulated except for PER1, which was downregulated by both test materials. Figure 2 shows the relationships between the regulated genes within the pathway and a summary of linear fold‐change data.

**FIGURE 3:**
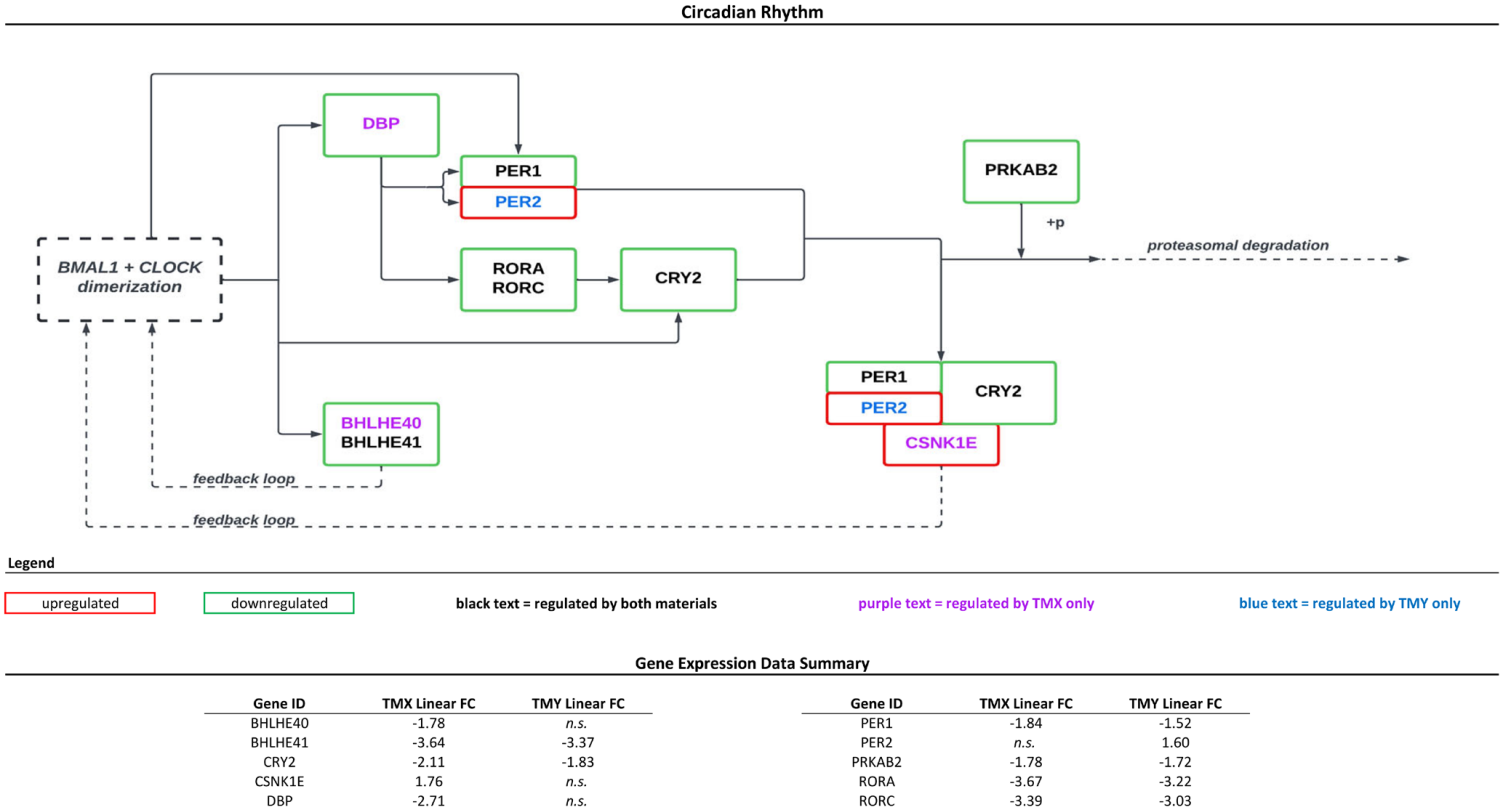
Circadian rhythm. Image adapted from KEGG submission for #04710, Kanehisa Laboratories, 02 August 2022. Only genes regulated in this study are shown; n.s., not significant. [Colour figure can be viewed at wileyonlinelibrary.com]

Both materials significantly regulated six genes for the Circadian Rhythm pathway, with ‘TMX’ regulating three unique genes and ‘TMY’ regulating one additional gene. All genes in this pathway were downregulated except for CSNK1E (upregulated by both materials) and PER2 (upregulated by ‘TMY’ only). The relationships between the regulated genes within the pathway and a summary of linear fold‐change data are shown in Figure 3.

Overall, ‘TMX’ and ‘TMY’ resulted in similar changes in gene expression related to circadian processes. Due to the regulation of different receptors (‘TMX’ via CACNA1G and ‘TMY’ via GRIN2A), the two materials appear to initiate changes in circadian entrainment through slightly different pathways but result in the same downstream effects (downregulation of several circadian rhythm‐associated genes). ‘TMY’ also resulted in the upregulation of PER2, which was not significantly regulated by ‘TMX’.

While the mechanisms involved in the regulation of circadian rhythm in the skin are well established, circadian entrainment is less well known. Entrainment, upstream of the circadian rhythm pathway, involves the binding of receptors such as calcium channels and glutamate‐gated ion channels. Signalling triggers such as the influx of calcium result in the activation of multiple additional cascade proteins, including calmodulins (CALM1), CamKII (CAMK2G), nNOS (NOS1), G‐proteins (GNG2, 4, 5, 10 and 11) and guanylate cyclases (GUCY1A1 and 1B1). The result of activating this cascade is the regulation of CREB, which in turn regulates core clock genes such as PER1, PER2 and FOS.

In addition to genes involved in circadian entrainment, core circadian rhythm genes were also regulated by the product formulations tested in this study. Core clock genes include BMAL1, CLOCK, NPAS2, PER1/PER2, CRY1/CRY2, ROR, REV‐ERE and DBP, among others. These molecules activate and then act upon each other, resulting in negative feedback loops that generate an approximately 24‐h rhythm. In this study, multiple core clock genes were downregulated (as opposed to multiple entrainment genes being upregulated). Downregulated clock genes included ROR (RORA and RORC), DBP, PER1 and CRY2. PER2 and CSNK1E were the only upregulated genes within the circadian rhythm pathway.

Of particular interest is that many of the genes associated with circadian entrainment were upregulated, while the genes involved with circadian rhythm were downregulated. This supports the cyclical and dependent nature of the specific pathway targets. In this study, gene expression was assessed at only a single time point. Analysis at additional time points may likely show changes in the opposite direction.

The results are consistent with other studies that have reported the regulation of circadian rhythm pathways in response to retinol and other topical cosmetic ingredients. Retinol has been shown to influence core clock genes such as BMAL1 and PER1 [39], and resveratrol regulates BMAL1 and CRY1 [40]. Several studies have shown that the endocannabinoid system can regulate circadian physiology [41]. Topical application of CBD alone and with other ingredients has been shown to regulate known clock genes, such as CIRT, CLOCK, CRY1 and PER2 [42].

### qPCR verification of select targets

Eleven (11) gene targets that were identified in the first set of tissues via RNASeq analysis were selected for verification via qPCR. In addition to the 48‐h time point analysed using the first set of tissues, additional tissues were also collected at 32 and 40 h to investigate the cyclical nature of these targets.

At the 48‐h time point, five out of the 11 genes were consistently regulated for both TMs (CRY2, GNG11, PER1, PRKG2 and RORA), one additional gene in ‘TMX’ and not ‘TMY’ (CACNA1G) and one additional gene in ‘TMY’ but not ‘TMX’ (NOS1), are shown in Table 1a,b.

**TABLE 1:** RNAseq and qPCR comparison. (a) TMX. (b) TMY. [Colour table can be viewed at wileyonlinelibrary.com]

Gene ID	log_2_FC		Avg. CT (qPCR)
	RNAseq	qPCR	
*(a) TMX versus saline control, 48 h*			
CACNA1G	1.93	4.77	27.137
CALM1	1.60	n.s.	25.414
CRY2	−2.11	−1.46	26.772
FOS	2.01	n.s.	34.194
GNG11	3.30	5.92	23.285
GRIN2A	n.s.	5.57	30.804
NOS1	1.99	n.s.	30.888
PER1	−1.84	−1.51	24.436
PRKG2	2.16	3.34	30.081
RORA	−3.67	−1.99	24.133
RYR1	6.67	n.s.	34.964
*(b) TMY versus saline control, 48 h*			
CACNA1G	n.s.	3.53	27.137
CALM1	1.59	n.s.	25.414
CRY2	−1.83	−1.39	26.772
FOS	2.05	n.s.	34.194
GNG11	2.58	3.98	23.285
GRIN2A	3.78	n.s.	30.804
NOS1	n.s.	n.s.	30.888
PER1	−1.52	−1.71	24.436
PRKG2	2.47	3.64	30.081
RORA	−3.22	−2.99	24.133
RYR1	7.62	n.s.	34.964

*Note*: Red text indicates genes which amplified above cycle 30. Green shading indicates gene regulation matched between methodologies.

Abbreviation: n.s., not statistically significant.

Most of the genes that were not consistently regulated between the two methodologies had qPCR cycle threshold (CT) values >30.0, indicating low expression of these transcripts in the sample tissues. Low levels of target transcript often result in increased variability and lower statistical significance, which likely accounts for the discrepancy between the two methods.

### Analysis of time points

**FIGURE 4:**
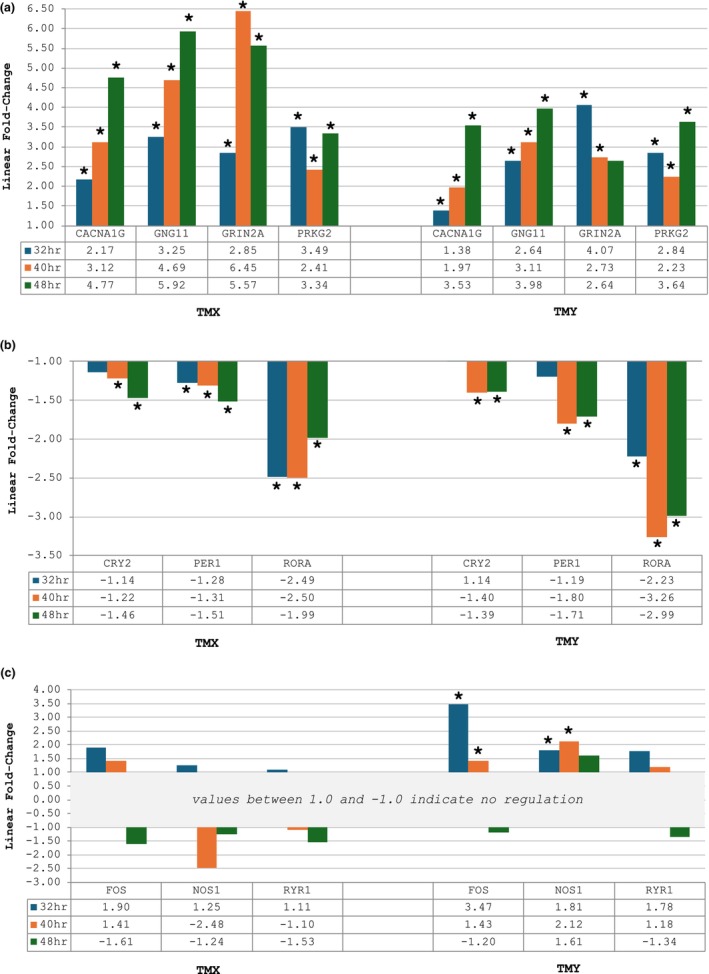
Gene regulation by TMX and TMY at 32, 40 and 48 h. (a) Upregulated genes; (b) Downregulated genes; (c) Genes which showed cycling, that is, early upregulation and later downregulation. * indicates significance (*p* < 0.05) versus time‐matched saline control. [Colour figure can be viewed at wileyonlinelibrary.com]

When comparing the qPCR gene expression results at 32, 40 and 48 h post‐exposure, several genes were regulated at each time point, as shown below in Figure 4a–c. For TMX, statistically significant (*p* < 0.05) upregulation was seen in CACNA1G, GNG11, GRIN2A and PRKG2 at all three time points. CRY2 and PER1 were significantly downregulated at 40 and 48 h, while RORA was significantly downregulated at all three time points. FOS, NOS1 and RYR1 showed early upregulation that switched to downregulation by 48 h; however, these fold changes were not significant.

For ‘TMY’, CACNA1G, GNG11 and PRKG2 were also significantly upregulated at all three time points; GRIN2A was upregulated at both 32 and 40 h. Like ‘TMX’, ‘TMY’ significantly downregulated CRY2 and PER1 at 40 and 48 h and RORA at all three time points. Cycling was also potentially observed in ‘TMY’, where FOS and NOS1 were significantly upregulated at earlier time points and not significantly regulated at 48 h. RYR1, while showing early upregulation and later downregulation, was again not statistically significant. CALM1 was not significantly up‐ or downregulated by either TM at any time point.

Based on the 48‐h RNA‐Seq data showing upregulation of several circadian entrainment genes and downregulation of several circadian rhythm targets, more cycling (i.e. switching between up and downregulation) of these targets at different time points in the qPCR analysis was expected. Since circadian processes cycle on a roughly 24‐h rhythm, time points within a 24‐h window (32, 40 and 48 h) were selected (32 and 40 h).

Lack of cycling in the qPCR data may have been impacted by the in vitro tissue model and the fact that the cultures were maintained throughout the study in standard cell culture conditions, that is, a dark environment with no light exposure. It is likely that individuals using the test product under ‘real world’ living conditions that include normal day–night schedules would exhibit changes in the entrainment and circadian genes in a more cyclic manner.

A potential future study may include human subjects or an altered culture method that includes timed exposure to light sources. Other circadian clock ‘synchronizers’ have been utilized in in vitro cell culture, such as thermal oscillations, short‐term exposure to serum (‘serum shock’) or exposure to dexamethasone [43, 44, 45], which could potentially be applied to this 3D tissue model in a future study to induce synchronized circadian cycling over longer treatment periods.

### Immunofluorescence

Protein targets for IF staining were selected based on multiple criteria, including significant gene regulation by at least one TM (preferably both), involvement in the entrainment pathway, availability of antibodies, etc. After a review of these selection criteria, the following five targets were processed for IF analysis: CALM1, GNG11, GUCY1A1, c‐FOS and PER2.

When compared to the saline vehicle control, GNG11 and c‐FOS showed downregulation by both ‘TMX’ and ‘TMY’, while CALM1 and GUCY1A1 showed upregulation by both materials. PER2 was upregulated by ‘TMY’ only.

**FIGURE 5:**
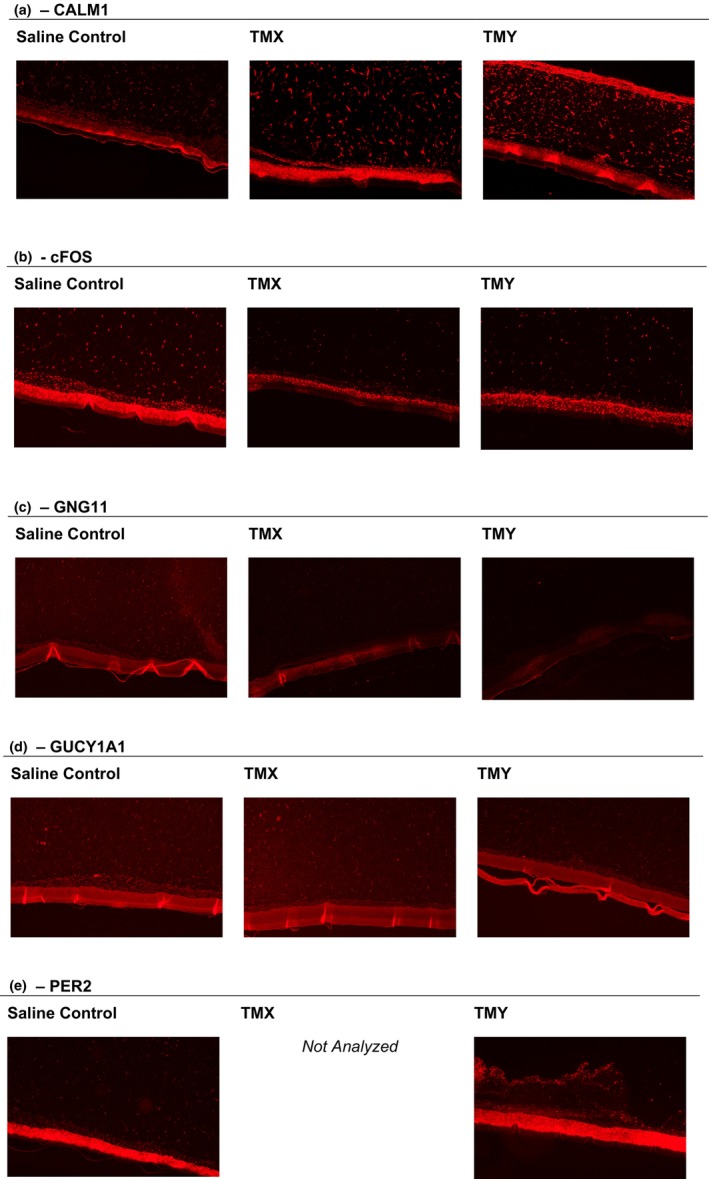
(a) CALM1. (b) cFOS. (c) GNG11. (d) GUCY1A1. (e) PER2. [Colour figure can be viewed at wileyonlinelibrary.com]

Figure 5a–e below show example visuals for each treatment group for each target.

Of the five proteins analysed for IF, protein expression for three targets was consistent with the gene expression results (CALM1, GUCY1A1 and PER2). GNG11 and c‐FOS were both upregulated in the gene expression data and downregulated via IF staining at the same time point (48 h post‐exposure; Table 2).

**TABLE 2:** Comparison of gene expression and immunofluorescence analyses.

Analysis	Test material	Target								
		CALM1	→	GUCY1A1	→	GNG11	→	FOS	→	PER2
Gene expression	TMX	UP		UP		UP		UP		n.s.
	TMY	UP		UP		UP		UP		UP
Immunofluorescence	TMX	UP		UP		DOWN		DOWN		N/A
	TMY	UP		UP		DOWN		DOWN		UP

Abbreviations: n.s., not significant; N/A, not analysed.

A discrepancy between gene expression and protein expression at the same time point can be expected, as changes in protein levels within a tissue may lag several hours behind the changes in mRNA expression. This is due to the time that it takes for translation from mRNA to protein to occur and has been observed in other studies [46].

## CONCLUSION

Circadian processes in the skin are related to, and may influence, many skin functions, including inflammatory response, wound healing and skin ageing. This study provided a ‘first step’ in determining the effects that ‘TMX’ and ‘TMY’ have on regulating the skin's circadian rhythm, with regulation via different receptors and pathways. Next steps may include analyses of specific downstream effects, including the inflammatory response in the skin, pathways involved in wound healing (i.e. cell proliferation and tissue remodelling) and potential anti‐ageing effects (i.e. skin elasticity and pigmentation). The results obtained in this study, including the regulation of 2000+ genes, indicate the potential of these formulations as effective skincare products.
